# Inhibition of Hedgehog-dependent tumors and cancer stem cells by a newly identified naturally occurring chemotype

**DOI:** 10.1038/cddis.2016.195

**Published:** 2016-09-22

**Authors:** Paola Infante, Romina Alfonsi, Cinzia Ingallina, Deborah Quaglio, Francesca Ghirga, Ilaria D'Acquarica, Flavia Bernardi, Laura Di Magno, Gianluca Canettieri, Isabella Screpanti, Alberto Gulino, Bruno Botta, Mattia Mori, Lucia Di Marcotullio

**Affiliations:** 1 Center for Life Nano Science@Sapienza, Istituto Italiano di Tecnologia, Viale Regina Elena 291 Rome, Italy; 2 Department of Molecular Medicine, Sapienza Università di Roma, Viale Regina Elena 291, Rome, Italy; 3 Dipartimento di Chimica e Tecnologie del Farmaco, Sapienza Università di Roma, Piazzale Aldo Moro 5, Rome, Italy; 4 Istituto Pasteur Fondazione Cenci Bolognetti, Sapienza Università di Roma, Viale Regina Elena 291, Rome, Italy

## Abstract

Hedgehog (Hh) inhibitors have emerged as valid tools in the treatment of a wide range of cancers. Indeed, aberrant activation of the Hh pathway occurring either by ligand-dependent or -independent mechanisms is a key driver in tumorigenesis. The smoothened (Smo) receptor is one of the main upstream transducers of the Hh signaling and is a validated target for the development of anticancer compounds, as underlined by the FDA-approved Smo antagonist Vismodegib (GDC-0449/Erivedge) for the treatment of basal cell carcinoma. However, Smo mutations that confer constitutive activity and drug resistance have emerged during treatment with Vismodegib. For this reason, the development of new effective Hh inhibitors represents a major challenge for cancer therapy. Natural products have always represented a unique source of lead structures in drug discovery, and in recent years have been used to modulate the Hh pathway at multiple levels. Here, starting from an *in house* library of natural compounds and their derivatives, we discovered novel chemotypes of Hh inhibitors by mean of virtual screening against the crystallographic structure of Smo. Hh functional based assay identified the chalcone derivative **12** as the most effective Hh inhibitor within the test set. The chalcone **12** binds the Smo receptor and promotes the displacement of Bodipy-Cyclopamine in both Smo WT and drug-resistant Smo mutant. Our molecule stands as a promising Smo antagonist able to specifically impair the growth of Hh-dependent tumor cells *in vitro* and *in vivo* and medulloblastoma stem-like cells and potentially overcome the associated drug resistance.

Hedgehog (Hh) signaling is a morphogenetic pathway that has a crucial role during embryonic development and tissues homeostasis.^1, 2, 3^ In vertebrates, Hh pathway activation is mediated by two transmembrane receptors: Patched1 (Ptch1), endowed with inhibitory functions, and Smoothened (Smo), which is the central transducer of Hh pathway and belongs to the class F (Frizzled) G protein-coupled receptor family. In physiological conditions, extracellular Hh ligand (Shh, Ihh, Dhh) binding to Ptch1 protein relieves its repression to Smo allowing signal transduction and activation of the Gli transcription factors, which in turn upregulate target genes involved in the most important cellular processes. Aberrant activation of Hh signaling is deeply involved in tumorigenesis. Indeed, activating germline or somatic mutations of genes encoding Hh pathway components are found in human and murine basal cell carcinoma (BCC) and medulloblastoma (MB).^4, 5^ Moreover, uncontrolled Hh signaling has been reported to drive tumor progression in several cancers, including lung, breast, stomach, pancreas and hematopoietic malignancies.^6^ For this reason, the development of Hh inhibitors is eliciting great interest in drug discovery. Vismodegib (GDC-0449/Erivedge) and others Smo antagonists have shown promising results in MB and BCC tumors. However, despite an initial clinical response, a number of drug-resistant Smo mutations were observed in patients also in recent clinical trials.^7, 8, 9^ Further, some clinical trials have failed so far,^10, 11, 12, 13^ due to poor pharmacokinetics, low selectivity on cancer stem cells (CSCs), and the presence of bystander co-regulatory mechanisms of the Hh pathway. Indeed, anti-Smo resistance is mediated by hyperactivation of the powerful downstream Gli factors due to Gli2 amplification during Vismodegib or Sonidegib (LDE-225) treatment,^4, 14^ or upregulation of Gli via a non-canonical Hh signaling activation, such as the induction of phosphoinositide 3-kinase (PI3K) pathway observed during Sonidegib administration.^15, 16^ Notably, non-canonical Hh mysregulation can also occur through Gli-independent events that include Src kinase activation,^17^ calcium spike activity at the primary cilium,^18^ activation of the GTPases Rac1 and RhoA by coupling of Smo to Gi proteins,^19^ and metabolic reprogramming by cilium-dependent Smo-Ca^2+^-AMPK axis.^20^ These findings raise the need for new effective Smo antagonists able to escape drug resistance and to counteract tumor growth.

Natural products are a unique source of remedies and medicines since ancient times, and still have a key role in modern drug discovery.^21, 22, 23^ The first Hh inhibitor ever discovered has been Cyclopamine, an alkaloid isolated from *Veratrum californicum* that potently antagonizes Smo and has efficacy against Hh-dependent tumors.^24, 25^ In recent years, several natural products have been found to impact on Hh transduction by direct or indirect mechanisms.^26^ Of note, in our previous effort to identify small molecules targeting Gli1/DNA interaction, the isoflavone GlaB has been discovered.^27^ These evidences clearly indicate that natural products represent a profitable source of chemotypes to modulate the Hh pathway at multiple levels.

To this end, an *in house* library of natural compounds and their derivatives was screened *in silico* towards the crystallographic structure of the Smo bound to Cyclopamine.^28^ Hh functional based assay identified the chalcone **12** as the most effective Hh inhibitor within the test set. **12** binds to Smo, is not sensitive to drug-resistant Smo mutation, and shows anti-oncogenic activity promoting growth arrest of Hh driven tumor cells and primary MB cells from Ptch^+/−^ mice, and inhibiting MB stem-like cells self-renewal.

In summary, in this work we identified the chalcone **12**, and other small molecules, which represent novel natural products chemotypes of Hh inhibitors.

## Results

### Virtual screening

To identify natural products chemotypes of Smo antagonists, an *in house* library of natural and synthetic compounds was screened *in silico* against the crystallographic structure of Smo bound to Cyclopamine (PDB: 4O9R).^28^ Although being of relatively restricted dimensions, the library is endowed with a noticeable chemical diversity and lead-like features, and has been successfully used in some computer-based hits/leads discovery studies.^27, 29^ The FRED docking program (OpenEye) was used for carrying out docking simulations. Molecules were ranked according to the Chemgauss4 score, and the predicted binding mode of the top 20% molecules was visually inspected. Virtual hits fitting the antagonists binding site of Smo, and interacting with key residues highlighted by crystallographic studies (namely, N219, Y394, K395, R400 and E518)^28, 30, 31^ were deemed top priority. After a subsequent analysis of chemical diversity, molecules **1–17** (Table 1) were submitted to functional investigation.

### Chemistry

The potential Smo antagonists 1–17 identified in silico (see Table 1) show a noticeable range of chemical diversity and differ also for their source, which is either natural or synthetic. Most of them belong to the flavonoids family, which includes flavonol (namely, **1** and **17**), flavanone (namely, **2** and **15**), isoflavon (namely, **9**, **13** and **16**) and isoflavanone (namely, **11**) derivatives. There are three Diels-Alder type adducts (namely, **5**, **6** and **8**) and three chalcones (namely, **7**, **10** and **12**). One steroid-derived alkaloid (namely, **4**) and one triterpene (namely, **14**) complete the test set, together with a phenylpropanoid glycoside (namely, **3**). The molecular weights range from about 312 to 658 Da, the highest values corresponding to the glycosylated (e.g., **2**, **3** and **15**) and Diels-Alder type adducts (namely **5**, **6** and **8**). The source of **1**–**17** are collected in Table 1, together with the reference data.^32, 33, 34, 35, 36, 37, 38, 39, 40, 41, 42, 43, 44^ **7**, **10** and **12** are of synthetic origin but endowed with a natural scaffold, namely chalcone, and have been synthesized by Claisen–Schmidt reaction, following a slightly modified procedure developed by Professor Bargellini (1879–1963).^45, 46, 47^

### Identification of Smo antagonists: functional screening, predicted binding mode and theoretical affinity

The inhibitory properties of the potential Smo antagonists **1–17** were investigated in a luciferase reporter assay that is widely used for characterizing Hh inhibitors. NIH3T3 Shh-Light II cells, stably incorporating a Gli-responsive firefly luciferase reporter (Gli-RE),^25^ were treated with the synthetic Smo agonist SAG^24^ alone or in combination with the selected small molecules to evaluate their dose–response ability to suppress Hh pathway. At the maximum concentration of 30 *μ*M, compounds **1–5** revealed no activity (Supplementary Figure S1a), whereas **6**–**10** and **11**–**12** showed mild and high activity, respectively (Supplementary Figure S1b and S1c) with an IC_50_ range of 4–38 *μ*M (Supplementary Table S1). We excluded the possibility that inhibition activity in this assay was mediated by cytotoxicity because no decrease was shown in the luciferase assay control *Renilla* as observed instead for **13–17**.

**Figure 1:**
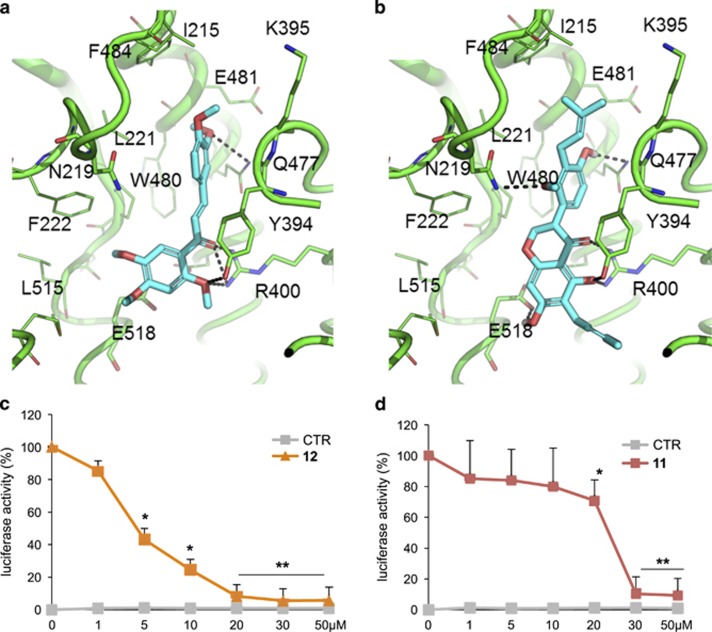
Inhibition of endogenous Hh signaling in NIH3T3 Shh-Light II cells and predicted binding mode to Smo. (**a** and **b**) Docking-based binding conformation of compounds **11** and **12** within the antagonists' site of Smo. The crystallographic structure of Smo encoded by PDB ID: 4O9R was used, and is showed as green cartoon. Residues within 5 Å from the ligands are showed as green lines. Residues involved in binding to well-known crystallographic Smo antagonists are highlighted as green sticks. Small molecules are showed as cyan sticks. H-bond interactions are highlighted by black dashed lines. (**c**–**d**) Dose–response curve of compound **12** (**c**) and compound **11** (**d**) in SAG-treated in comparison with untreated NIH3T3 Shh-Light II cells. Treatment time was 48 h, and normalization was against *Renilla* luciferase. Data show the mean±S.D. of three independent experiments. *P<0.05; **P<0.01 *versus* SAG

These data were in accordance with the molecular docking simulations showing that **6**–**12** and, particularly the most active **11** and **12** are able to fit the antagonists' site of Smo, which is located within its heptahelical bundle, and to establish interaction with its key residues. In particular, **11** and **12** bind into the hydrophobic pocket bounded by residues F484, I215, L221, M301, L303, W480, F222 and Y394. This latter residue also establishes π–π stacking interaction and an H-bond with **11** and **12** (Figures 1a and b). Additional H-bonds are established with, N219, Q477, E518 and R400. Notably, these residues have been already identified in X-ray crystallography studies as crucial for small molecules binding to Smo.^28, 30, 31^ Finally, the binding mode of **11** and **12** is highly comparable and show a noticeable shape and pharmacophoric overlapping each other. The predicted binding mode of molecules **6**–**10** is reported in Supplementary Figure S2, and is highly consistent with the above description, even if it is characterized by a fewer interactions with Smo residues, as well as with a worse score than **11** and **12** (Supplementary Table S1). For the sake of clarity, molecular docking was performed exclusively against the well-known antagonists site of Smo, occupied by the natural antagonist Cyclopamine in the selected X-ray structure.^28^ Indeed, our biological data convincingly support that Hh inhibitors **11** and **12** interact with Smo within the same site as Cyclopamine.^48^

**Figure 2:**
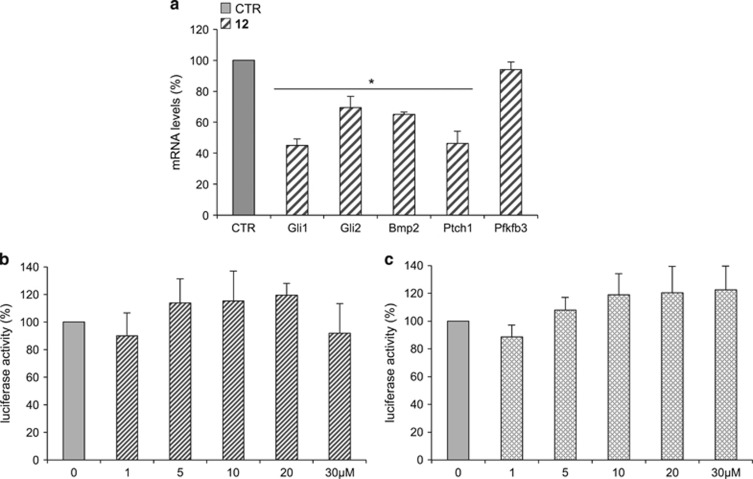
Compound **12** inhibits Hh signaling without affecting AP1/Jun and WNT/β-Catenin pathways. (**a**) The graphs show the Hh target genes expression levels in Ptch1^−/−^ MEFs treated for 48 h with **12** or DMSO as control. mRNA levels were determined by quantitative real-time PCR (qRT-PCR) normalized to endogenous control (β2-microglobulin and HPRT). Pfkfb3 gene was used as negative control. *P<0.05 *versus* CTR. (**b** and **c**) AP1/Jun and WNT/β-Catenin pathways activity were assayed in MEFs WT transfected with MMP1-luciferase reporter and c-Jun (**b**) or Top Flash-luciferase reporter and β-Catenin (**c**), respectively, in the presence or absence of increasing concentrations of compound **12**. Treatment time was 24 h, and normalization was against *Renilla* luciferase. Data show the mean±S.D. of three independent experiments

In more detail, a dose–response curve of **11** and **12** in NIH3T3 Shh-light II cells luciferase assay proved that **12** (2',4',5',3,4-pentamethoxychalcone) was the most powerful in inhibiting the Hh pathway, with an IC_50_ of 4.44 *μ*M (Figure 1c and Supplementary Table S1) while **11**, namely isosophoranone, showed a lower effect, with an IC_50_ value of 22.56 *μ*M (Figure 1d). For this reason, we focused further studies only on **12**. To investigate the inhibitory properties of **12** on Hh pathway, we analyzed endogenous Hh target gene activation in genetically defined Ptch1^−/−^ mouse embryonic fibroblasts (Ptch1^−/−^ MEFs) (Figure 2a). In these cells, constitutive activation of the Hh pathway is the consequence of the loss of repressive receptor *Ptch1* gene, thus determining high expression levels of Hh target. Compound **12**, at a concentration of 5 μM, significantly reduced mRNA levels of several endogenous Hh target genes, including Gli1, the most powerful effector of Hh signaling.^5^

Importantly, **12** revealed specificity of action for Hh signaling without affecting luciferase activity driven by Hh-unrelated (i.e., Jun/AP1) (Figure 2b) and Hh-related (i.e., Wnt/β-catenin) signaling pathways, respectively (Figure 2c).

Docking-based complexes were further relaxed through energy minimization in explicit solvent, and their theoretical affinity to Smo was estimated by means of a rescoring procedure based on multiple functions (Supplementary Table S1). The Chemgauss4 and the Chemscore functions proved to rank correctly the compounds and to discriminate quite well Hh inhibitors from inactives. In contrast, rescoring with XSCORE or the Molecular-Mechanics Generalized Born Surface Area (MM-GBSA) approach provided a worse ranking of tested compounds (Supplementary Table S1).^49, 50, 51^ This benchmarking study performed on diverse naturally occurring chemotypes may facilitate future structure-based virtual screenings against Smo.

It is worth mentioning that **12** was predicted not to interact with D473 (Supplementary Figure S3), a key residue responsible for drug resistance at the Smo receptor upon mutation to histidine (D473H), as identified in clinical patients treated with Vismodegib.^7^ Therefore, it is expected that this molecule could be active also against the drug-resistant form of the Smo receptor, thus representing a potential benefit for clinical applications.

### Compound 12 binding to cells expressing Smo wild-type or drug-resistant Smo mutant

**Figure 3:**
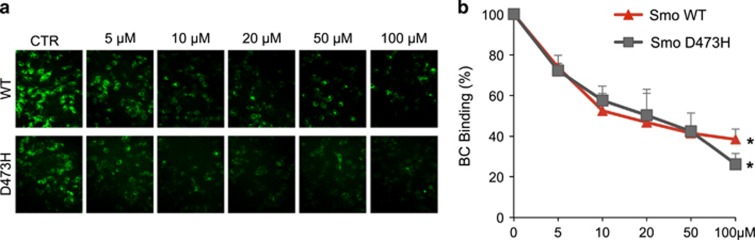
Compound **12** inhibition of Bodipy-Cyclopamine (BC) binding to whole cell expressing either Smo WT or Smo D473H. (**a**) Competitive binding of Bodipy-Cyclopamine (BC) in HEK293T cells transfected transiently with human WT or mutant Smo (D473H) was conducted with various concentrations of compound **12**. BC binding (green) is visualized using fluorescence microscopy in a representative field. (**b**) The concentrations–response curves express the percentage of BC incorporation observed after compound **12** treatment in HEK293T cells transfected with human WT or mutant (D473H) Smo, respectively. Data show the mean±S.D. of three independent experiments. *P<0.05 *versus* CTR. Quantitative data are the average BC intensity from five independent fluorescence microscopy images

To verify the direct action of compound **12** on Smo receptor, we carried out a displacement assay based on the use of the Bodipy-Cyclopamine (BC), a fluorescent derivative of Cyclopamine that interacts with Smo at the level of its heptahelical bundle.^28^ Moreover, we used this assay to verify the ability of **12** to bind both Smo WT and Smo D473H mutant, the first described human Smo point mutation, which confers resistance to treatment with the Smo antagonist Vismodegib.^7, 52^ Indeed, drug-resistance due to Smo mutations has raised the need to develop novel Smo antagonists able to overcome this main limitation for the clinical development of effective anti-Smo molecules.^7, 8, 9, 53^ To this end, HEK293T cells transfected with a vector expressing Smo WT or Smo D473H mutant, were incubated with BC in the absence or presence of various concentrations of **12**. As shown in Figures 3a and b, **12** revealed comparable effects on Smo WT and Smo D473H, showing dose-dependent effects and equal binding affinity corresponding to similar IC_50_ values (**12** on Smo WT IC_50_=24.50 *μ*M, and on Smo D473H IC_50_=22.68 *μ*M). Moreover, **12** significantly inhibited BC binding to murine Smo WT and the mouse orthologous Smo D477G mutant (Supplementary Figure S4). Notably, while Vismodegib showed about 1000-fold loss affinity for the resistant Smo mutants in binding assay, **12** conserved the same affinity (Supplementary Table S2). These *in vitro* findings reveal that **12** acts as Smo antagonist by binding within the Cyclopamine site, and suggest its potential use for the treatment of cancers that are dependent on Hh signaling, including Vismodegib-resistant tumors.

### Compound 12 inhibits Hh-dependent cell growth of cerebellar granule cell progenitors

**Figure 4:**
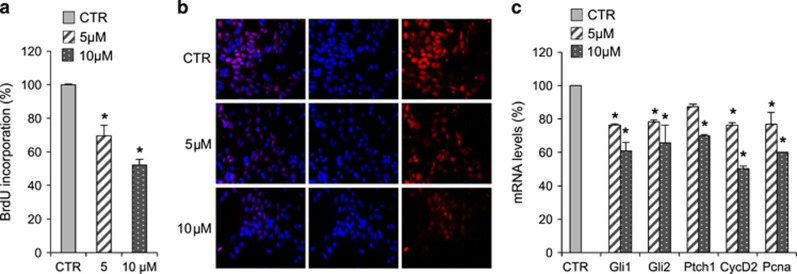
Compound **12** inhibition of Hh-dependent growth of cerebellum granule cell progenitors. (**a** and **b**) BrdU assay in cerebellar granule cell progenitors (GCPs). GCPs isolated from 4-day-old mice were treated with SAG alone or in combination with compound **12** at 5 and 10 *μ*M concentrations for 48 h. (**a**) Inhibition of cell proliferation was measured as percentage of BrdU incorporation in comparison with SAG-treated sample. Data show the mean±S.D. of three independent experiments. *P<0.05 SAG+compound **12** *versus* SAG (CTR). (**b**) The immunofluorescence staining of BrdU (red) and nuclear Hoechst staining (blue) show the decrease of BrdU uptake after compound **12** treatment (5 and 10 *μ*M). (**c**) qRT-PCR analysis show Hh and proliferation targets mRNA expression levels determined in GCPs culture derived from 4-day-old mouse cerebella treated with SAG alone or in combination with compound **12** at 5 and 10 *μ*M concentrations for 48 h. In all qRT-PCR experiments, the results were normalized to endogenous control (*β2-microglobulin* and *HPRT*). Shown is the mean of three independent experiments. Error bars indicate S.D. *P<0.05 SAG+compound **12** *versus* SAG (CTR)

Hh signaling is a critical regulator of cerebellum development controlling the proliferation of granule cell progenitors (GCPs) under Purkinje cell-derived Shh stimuli. Withdrawal of Hh signal, occurring physiologically after the first post-natal week in mice,^54^ causes cell growth arrest and induces their differentiation into mature granules.^55^ Importantly, genetic and epigenetic alterations in the Hh pathway are responsible for the lack of GCPs proliferation arrest, leading to the tumorigenic conversion of these progenitors, considered as the cell of origin of MB.^56, 57^ To investigate the biological effects of **12**, we first tested its ability to suppress Hh-dependent growth in 4-day-old mouse cerebellar progenitors. While treatment of GCPs with the Smo agonist SAG-enhanced cell BrdU uptake (Figures 4a and b), the addition of **12** reduced significantly this activity, decreasing the proliferation rate in a dose-dependent way (Figures 4a and b). Accordingly, GCPs treated with **12** displayed reduced mRNA levels of Hh target genes and markers related to cell growth (i.e., *Gli1*, *Gli2*, *Ptch1*, *Pcna*, *cyclin D2*) correlating the decrease of GCPs proliferation after **12** treatment with its inhibitory effects on Hh signature (Figure 4c).

### Compound 12 inhibits the growth of Hh-dependent tumor cells *in vitro* and *in vivo*

**Figure 5:**
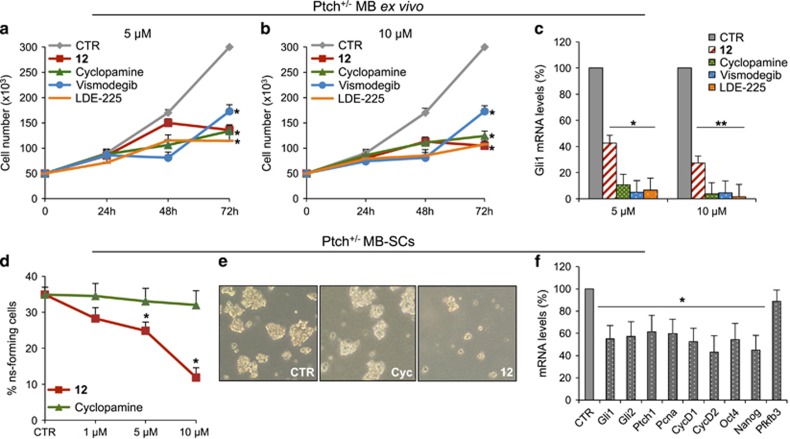
Compound **12** inhibition of Hh-dependent MB tumor cell growth. (**a**–**c**) *Ex vivo* cell cultures from Ptch1^+/−^ mice MBs were treated with compound **12**, Cyclopamine, Vismodegib, LDE-225 or DMSO only. (**a** and **b**) After the indicated times, a trypan blue count was performed to determine the growth rate of viable cells. (**c**) *Gli1* mRNA expression levels were determined by qRT-PCR normalized to endogenous control (*β2-microglobulin* and *HPRT*). (**d**–**f**) Compound **12** inhibits MB-SCs self-renewal. (**d**) Suspension of single MB-SCs isolated from Ptch1^+/−^ mice were cultured in stem cell medium to allow the formation of primary neurospheres. Primary neurospheres were dissociated and treated with increasing concentrations of compound **12,** Cyclopamine or DMSO only. After 7 days of treatment, the number of secondary neurospheres derived from a known number of single cells was counted. The self-renewal MB-SCs' capability is expressed as percentage of neurosphere-forming cells. (**e**) Representative bright-field images of tumor neurospheres after compound **12** or Cyclopamine treatment are also shown. (**f**) MB-SCs isolated from Ptch1^+/−^ mice MBs were treated for 48 h with compound **12** or DMSO only. qRT-PCR analysis show Hh, proliferation and stemness target mRNA. For qRT-PCR, results were normalized to endogenous control (*β2-microglobulin and HPRT*). All data show the mean±S.D. of three independent experiments. *P<0.05; **P<0.01 *versus* DMSO (CTR)

To verify the efficacy of **12** to suppress the proliferation of cancer cells in comparison with other Smo antagonists, we used Hh-dependent tumor cell models, such as MB, BCC and prostate cancer. Primary MB cells freshly isolated from Ptch1^+/−^ mice tumors^58, 59, 60^ and treated with **12** showed the significant inhibition of the proliferation in comparison with other Smo antagonists (namely, Cyclopamine, Vismodegib and LDE-225), as consequence of the decrease of *Gli1* mRNA levels (Figures 5a–c).

Hh signaling has a central role in stem/progenitor cell maintenance and self-renewal. In several tumors, including MB, the aberrant activation of Hh signaling contributes to CSCs proliferation by the Gli mediated regulation of stemness marker *Nanog*.^61, 62^ The presence of CSCs in the tumor mass is a major cause of resistance and favors tumor relapse,^63^ thus representing an attractive druggable targets for anticancer therapy. Therefore, CSCs appear appealing tools for testing the therapeutic potential of the **12** herein identified. We show that **12**, but not Cyclopamine, suppressed the clonogenic self-renewal ability of murine Ptch1^+/−^ MB stem-like cells to form spheres from single-cell suspension (Figure 5d) and they appeared reduced in number and size (Figure 5e). Consistent with these results, **12** reduced Hh pathway activity in MB stem-like cells as evaluated by the decrease of the pathway readouts *Gli1*, *Gli2* and *Ptch1* mRNAs, stemness markers (*Nanog*, *Oct4*) as well as growth genes expression (*cyclins D1* and *D2*, *Pcna*) (Figure 5f).

**Figure 6:**
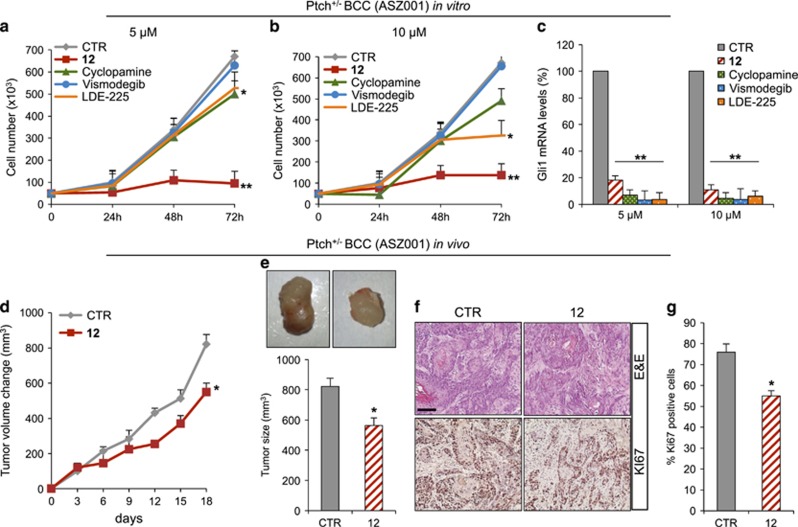
Compound **12** inhibition of Hh-dependent BCC cell growth *in vitro* and *in vivo*. (**a**–**c**) Compound **12** inhibition of Hh-dependent BCC tumor cell growth. ASZ001 BCC cells were treated with compound **12**, Cyclopamine, Vismodegib, LDE-225 or DMSO only (**a** and **b**). After the indicated times, a trypan blue count was performed to determine the growth rate. *Gli1* mRNA expression levels were determined by qRT-PCR after treatment of ASZ001 BCC cells with compound **12**, Cyclopamine, Vismodegib, LDE-225 or DMSO only (**c**). Results were normalized to endogenous control (*β2-microglobulin* and *HPRT*). All data show the mean±S.D. of three independent experiments. *P<0.05; **P<0.01 *versus* DMSO (CTR). (**d**–**g**) ASZ001 BCC allografts. Change of tumor volume during compound **12** or vehicle treatment period (**d**). Representative flank allografts average volumes (**e**). H&E and immunohistochemical staining of Ki67 of allograft tumor samples (**f**). Scale bars represent 250 *μ*m for H&E and Ki67. Quantification of Ki67 staining from immunohistochemistry shown in (**f**). (**g**) Shown is the mean±S.D. of tumor (*n*=6) for each treatment. *P<0.05 *versus* CTR

The ability of **12** to inhibit tumor cells proliferation was also tested in mouse ASZ001 BCC cells, previously characterized as a specific Hh-dependent tumor cell line harboring *Ptch1* deletion.^64^ **12** showed significant efficacy to impair ASZ001 BCC cell growth *in vitro* compared with Cyclopamine, Vismodegib and LDE-225 (Figures 6a and b), in agreement with the decrease of *Gli1* mRNA levels observed after drug treatment (Figure 6c). The inhibitory effect of **12** on tumor growth was also confirmed *in vivo* using a BCC allograft model. To this end, NOD/SCID mice were engrafted with ASZ001 BCC cells and treated every second day with s.c. injections of **12** at a concentration of 50 mg/kg or vehicle alone. During the treatment period, we observed reduction of tumor cell growth in **12** treated mice compared with the controls (Figures 6d and e), consistently with decreased percentage of Ki67 labeling (Figures 6f and g).

**Figure 7:**
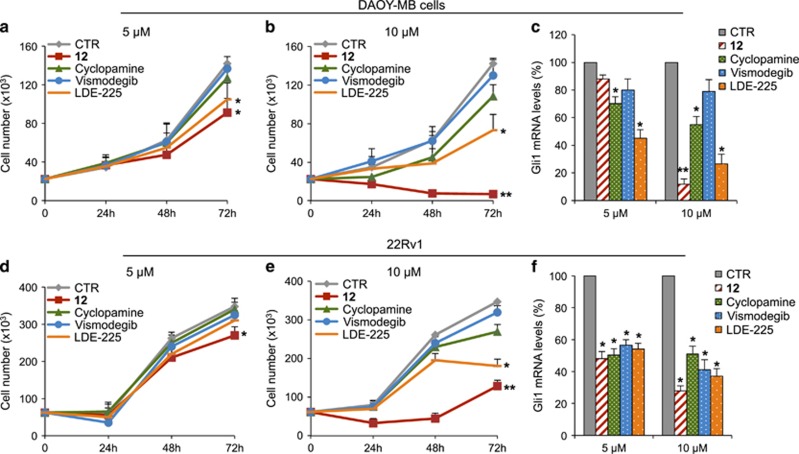
Compound **12** inhibition of human Hh-dependent tumor cell growth. Human medulloblastoma DAOY (**a** and **b**) or human prostate carcinoma epithelial 22Rv1 cells (**d** and **e**) were treated with compound **12**, Cyclopamine, Vismodegib, LDE-225 or DMSO only. After the indicated times, a trypan blue count was performed to determine the growth rate. *Gli1* mRNA expression levels were determined by qRT-PCR after treatment of DAOY (**c**) or 22Rv1 (**f**) cells with compound **12**, Cyclopamine, Vismodegib, LDE-225 or DMSO only. Results were normalized to endogenous control (*β-actin* and *HPRT*). All data show the mean±S.D. of three independent experiments. *P<0.05; **P<0.01 *versus* DMSO (CTR)

To elucidate the anti-proliferative effects of **12** in human cancer cell lines,^65, 66, 67^ we investigated its ability to block the growth of MB, BCC and prostate cancer cells, which are convenient model for monitoring the pharmacological inhibition of the Hh pathway.^68, 69^ As shown in Figure 7, **12** displayed an higher activity than other Smo antagonists in inhibiting DAOY and 22Rv1 cell proliferation (Figures 7a and b, d and e), consistent with the significant decrease of *Gli1* mRNA levels after treatment (Figures 7c and f). Similar results were obtained in human BCC TE354T cell line, previously described as Hh-dependent cells,^70^ in which **12** was compared with Vismodegib (Supplementary Figure S5). Overall, these results confirmed that **12** inhibits the proliferation of Hh-driven tumor cells.

## Discussion

In this study, we identified novel naturally occurring chemotypes of Hh inhibitors by mean of an integrated multidisciplinary study mixing molecular modeling with chemistry, molecular and cell biology. Particularly, the 2',4',5',3,4-pentamethoxychalcone (**12)**, was the most potent Hh inhibitor identified herein, and proved to impair the growth of Hh-dependent tumor cells *in vitro* and *in vivo*.

In this study, we also pinpointed the relevance of natural products as useful source for drug discovery in cancer therapy. In fact, natural products represent more than one-third of all FDA-approved new molecular entities, and one-quarter of these derived from plants.^21^ Near the end of the Twentieth century, the use of natural products seriously declined in favor of new emerging technologies to generate drug candidates. However, these strategies did not deliver the expected results and recently there has been a renewed interest in the use of natural products.^23, 41, 71, 72^ Particularly, chemistry has emerged as the preferred tool to modify natural products up to suitable drug candidates, as underlined by the Cyclopamine derivative IPI–926, which has recently completed phase II clinical trials.^73^

Cancer is one of the main human diseases for which natural products find therapeutic applications. In this context, the morphogenetic Hh signaling represents a noticeable example of druggable tumorigenic pathway. In the past years, many research efforts have been spent in the identification of Hh antagonists able to suppress the aberrant activation of this signaling occurred in several disparate human tumors. The first Hh inhibitor ever discovered has been Cyclopamine, an alkaloid isolated from *Veratrum californicum* that antagonizes Smo and has efficacy against Hh-dependent tumors.^25^ Moreover, the isoflavone genistein, first isolated in *Genista tinctoria* and naturally occurring in several plants including tobacco and maize, inhibits weakly the Hh pathway.^74^ Other natural products have been found to impair Hh transduction by acting on the main positive regulators of this signaling, both upstream on Smo^69^ and downstream on the transcription factor Gli1.^12, 27^

Recently, the clinical development of Smo antagonists has proved disappointing because their scarce pharmacokinetics, severe side effects, and the emergence of drug resistance due to point mutations that rendered Smo insensitive to the drugs. With the aim to identify natural products as new Smo antagonists able to overcome these limits, we performed a docking-based virtual screening of an *in house* library of natural compounds and their derivatives, composed of about 1200 small molecules of natural or synthetic origin. This structure-based approach significantly beneficed of the crystallographic structure of Smo bound to the natural antagonist Cyclopamine. Seventeen compounds selected *in silico* were subsequently screened for their ability to counteract Hh activity by a luciferase functional assay in NIH3T3 Shh-Light II cells. According to this *in vitro* assay, five compounds (**1–5**) were inactive, five (**6**–**10**) showed mild activity and two (**11–12**) revealed high activity, whereas **13–17** resulted toxic. The dose–response activity of chalcone **12**, the most effective molecule here identified, was supported by the demonstration of its direct binding to Smo receptor, as predicted by molecular modeling and confirmed in a displacement assay with BC. More interestingly, **12** resulted active on the D473H drug-resistant Smo mutant, the main cause of failure of the Vismodegib treatment, suggesting the possible therapeutic applicability of **12** for the treatment of Vismodegib-resistant tumors.^75^ The strong effect of **12** to inhibit Hh activity compared with BC displacement assay supposed its ability to affect other positive regulators of the Hh signaling. In this regard, **12** suppressed the expression of endogenous Hh target genes in Ptch1^−/−^, as well as in SuFu^−/−^ MEFs (Supplementary Figure S6),^76^ two cell models in which the constitutive activation of Hh signaling is consequence of the genetic ablations of the upstream Ptch1 and the downstream SuFu-negative regulators, respectively.

Hh inhibition by **12** was also observed in the physiological cellular context of GCPs, whose proliferation is under Hh pathway controls during the cerebellum development. Indeed, **12** suppressed Hh gene signature in SAG-treated GCPs cells, with the consequent decrease of their proliferation. Noteworthy, **12** demonstrated specificity of action for Hh signaling, resulting inactive on both Hh-related and -unrelated pathways, such as Wnt/β-catenin and Jun/AP1 signaling, respectively. Aberrant activation of Hh pathway leads to tumorigenesis by ligand-independent mutational activation of the Hh signaling or ligand-dependent paracrine signaling.^77^ In the paracrine model of Hh-dependent oncogenesis, the ligand produced by tumor-derived cells signals to the surrounding stromal cells and indirectly promotes tumor growth. Here, we demonstrate the ability of **12** to selectively inhibit the *Gli1* mRNA expression and the proliferation of Hh-dependent tumor cells *in vitro* and *in vivo*, including MB and BCC, two tumor models where Hh pathway activity occurs by ligand-independent manner. The effect of our compound in tumor models displaying ligand-dependent Hh pathway activation such as colorectal, pancreatic and bladder cancer, would be interesting to be investigated.

Overall, our findings underline the relevance of natural products as useful source for drug discovery in cancer therapy, and discover a new specific Smo antagonist that inhibits the Hh-dependent tumor cells growth, and stands as a valuable starting point to develop potential therapeutic agents for Vismodegib- or Sonidegib-resistant tumors.

## Experimental section

### Chemistry

Source of compounds **1**–**17**. All the tested compounds (namely, **1–17**) are known structures which belong to our *in house* library of natural products. Chemical identity of compounds **1–17** was assessed by re-running NMR experiments, which proved to be in agreement with the literature data reported below for each compound. The purity of all compounds, checked by reversed-phase HPLC under the chromatographic conditions reported in the Supporting Information, was always higher than 95%.

Compound **1** (myricetin or 3,5,7-trihydroxy-2-(3,4,5-trihydroxy-phenyl)-4H-chromen-4-one) was purchased from Sigma Aldrich, St. Louis, MO, USA, as used without further modification.

Compound **2** (naringin or (2*S*)-7-[(2*S*,3*R*,4*S*,5*S*,6*R*)-4,5-dihydroxy-6-(hydroxymethyl)-3-[(2*S*,3*R*,4*R*,5*R*,6*S*)-3,4,5-trihydroxy-6-methyloxan-2-yl]oxyoxan-2-yl]oxy-5-hydroxy-2-(4-hydroxyphenyl)-2,3-dihydrochromen-4-one was purchased from Sigma-Aldrich, as used without further modification.

Compound **3** (martinoside or [(2*R*,3*R*,4*R*,5*R*,6*R*)-5-hydroxy-6-[2-(3-hydroxy-4-methoxyphenyl)ethoxy]-2-(hydroxymethyl)-4-[(2*S*,3*R*,4*R*,5*R*,6*S*)-3,4,5-trihydroxy-6-methyloxan-2-yl]oxyoxan-3-yl] (*E*)-3-(4-hydroxy-3-methoxyphenyl)prop-2-enoate) showed NMR spectra identical to the literature.^41^

Compound **4** (cevadine or (3β,4α,9β,16β)-4,12,14,16,17,20-hexahydroxy-4,9-epoxycevan-3-yl (2*Z*)-2-methyl-2-butenoate) showed NMR spectra identical to the literature.^78^

Compound **5** (sorocein B or (2*E*)-1-[(3a*S*,13b*S*,13c*R*)-4,11-dihydroxy-8a-(5-hydroxy-2,2-dimethyl-2H-chromen-8-yl)-2-methyl-1,8a,13b,13c-tetrahydro-3aH-benzo[3,4]isochromeno[1,8-bc]chromen-5-yl]-3-(2,4-dihydroxyphenyl)-2-propen-1-one) showed NMR spectra identical to the literature.^43^

Compound **6** (sorocein A or (3a*S*,13b*S*,13c*R*)-6-[(*E*)-2-(2,4-dihydroxyphenyl)vinyl]-8a-(5-hydroxy-2,2-dimethyl-2H-chromen-8-yl)-2-methyl-1,8a,13b,13c-tetrahydro-3aH-benzo[3,4]isochromeno[1,8-bc]chromene-4,11-diol) showed NMR spectra identical to the literature.^43^

Compound **7** (dihydrochalcone or 3-benzo[1,3]dioxol-5-yl-1-(2,4,5-trimethoxy-phenyl)-propan-1-one) showed NMR spectra identical to the literature.^79^

Compound **8** (kuwanol E or [2,4-dihydroxy-3-(3-methyl-2-buten-1-yl)phenyl][(1*R*,2*S*,6*S*)-6-(2,4-dihydroxyphenyl)-2-{4-[(*E*)-2-(2,4-dihydroxyphenyl)vinyl]-2,6-dihydroxyphenyl}-4-methyl-3-cyclohexen-1-yl]methanone) showed NMR spectra identical to the literature.^80^

Compound **9** (derrustone or 3-(1,3-benzodioxol-5-yl)-5,7-dimethoxy-4H-chromen-4-one) showed NMR spectra identical to the literature.^81^

Compound **10** (2',3',4',6',3,4-hexamethoxychalcone or 3-(3,4-dimethoxy-phenyl)-1-(2,3,4,6-tetramethoxy-phenyl)-2-propen-1-one) showed NMR spectra identical to the literature.^79^

Compound **11** (isosophoranone or 5,7-dihydroxy-3-[4-hydroxy-2-methoxy-3-(3-methyl-2-buten-1-yl)phenyl]-6-(3-methyl-2-buten-1-yl)-2,3-dihydro-4H-chromen-4-one) showed NMR spectra identical to the literature.^82, 83, 84^

Compound **12** (2',4',5',3,4-pentamethoxychalcone or 3-(3,4-dimethoxy-phenyl)-1-(2,4,5-trimethoxy-phenyl)-2-propen-1-one) showed NMR spectra identical to the literature.^79^

Compound **13** (auriculasin or 7-(3,4-dihydroxyphenyl)-5-hydroxy-2,2-dimethyl-10-(3-methyl-2-buten-1-yl)-2H,6H-pyrano[3,2-g]chromen-6-one) showed NMR spectra identical to the literature.^35^

Compound **14** (barbinervic acid or (1*R*,2*R*,4a*S*,6a*R*,6a*S*,6b*R*,8a*R*,9*S*,10*R*,12a*R*,14b*S*)-1,10-dihydroxy-9-(hydroxymethyl)-1,2,6a,6b,9,12a-hexamethyl-2,3,4,5,6,6a,7,8,8a,10,11,12,13,14b-tetradecahydropicene-4a-carboxylic acid) showed NMR spectra identical to the literature.^85^

Compound **15** (hesperidin or (2*S*)-5-hydroxy-2-(3-hydroxy-4-methoxyphenyl)-7-[(2*S*,3*R*,4*S*,5*S*,6*R*)-3,4,5-trihydroxy-6-[[(2*R*,3*R*,4*R*,5*R*,6*S*)-3,4,5-trihydroxy-6-methyloxan-2-yl]oxymethyl]oxan-2-yl]oxy-2,3-dihydrochromen-4-one) was purchased from Sigma-Aldrich, as used without further modification.

Compound **16** (cabreuvin or 3-(3,4-dimethoxyphenyl)-7-methoxy-4H-chromen-4-one) showed NMR spectra identical to the literature.^33^

Compound **17** (jaceidin or 5,7-dihydroxy-2-(4-hydroxy-3-methoxyphenyl)-3,6-dimethoxy-4H-chromen-4-one) showed NMR spectra identical to the literature.^32^

### Molecular modeling

The main features of the *in house* library of natural compounds and their derivatives have been already described elsewhere.^27, 29^ Conformational analysis was performed with OMEGA2 from OpenEye,^86, 87^ keeping all parameters at their default values and allowing the storage of up to 600 conformers of each molecule in the final database. Hydrogen sampling options were activated. The receptor for molecular docking simulations was prepared with the *make_receptor* utility of OEDocking. Molecular docking was performed with the FRED docking program (OpenEye),^88, 89, 90^ using the highest-resolution settings. During virtual screening, only the best pose of each compound was saved and virtual Smo antagonists were ranked according to the Chemgauss4 score. In subsequent accurate docking simulations, up to 10 poses of each molecule were stored. Energy minimization of docking complexed was performed with Amber12.^91^ In detail, Smo/ligand complexes were solvated in a rectilinear box of TIP3P typed water molecules buffering 4Å from the protein. The solvent was first energy minimized for 100 steps with the Steepest Descent algorithm (S.D.) and for subsequent 200 steps with the Conjugate Gradient algorithm (CG) before to energy minimize the solvated solute for 1000 steps S.D. and subsequent 4000 steps CG.

Rescoring was performed with multiple programs and functions including XSCORE,^49^ Chemscore,^92^ and the MM GBSA method implemented in Amber12, using settings previously described.^50, 91, 93^

### Biology

#### Cell cultures, transfection and treatments

HEK293T, Shh-Light II, Ptch1^–/–^ MEFs, SuFu^–/–^ MEFs, wild-type MEFs and 22Rv1 cells were cultured in DMEM plus 10% FBS. DAOY cells were maintained in Eagle's Minimum Essential Medium (MEM) plus 10% FBS. All media contained l–glutamine and antibiotics. ASZ001 BCC cells were cultured in 154CF medium (Gibco-BRL, Grand Island, NY, USA) plus 2% FBS chelated with Chelex 100 sodium form (Sigma Aldrich), calcium chloride 0.05 mM (Gibco-BRL) and antibiotics. Cerebellar GCPs (from 4-days-old mice) were isolated and cultured as previously described.^94^ TE354T human BCC cells (ATCC CRL–7762) were cultured in DMEM medium (ATCC 30–2002) plus 10% FBS and antibiotics. Murine MBs were isolated from Ptch1^+/–^ mice (The Jackson Laboratory, Bar Harbor, ME, USA). Tissues were collected as previously described,^27^ and immediately prepared cell suspensions were used for short-term cultures to keep Hh-sensitivity *in vitro*.^58, 59, 60^ Transient transfections were performed using DreamFect^TM^ Gold transfection reagent (Oz Biosciences SAS, Marseille, France). Cells were treated with SAG (200 nM, Alexis Biochemicals Farmingdale, NY, USA), Bodipy-Cyclopamine (5 nM, BioVision Inc., San Francisco, CA, USA), Cyclopamine (Calbiochem, Nottingham, UK), Vismodegib (Selleckchem, Munich, Germany), LDE-225 (Selleckchem, Munich, Germany).

#### Hh-dependent luciferase reporter assay

The luciferase assay was performed in Shh-Light II cells, stably incorporating a Gli-responsive luciferase reporter and the pRL-TK Renilla (normalization control), treated for 48 h with SAG (200 nM) and the studied compounds. Luciferase and *Renilla* activity were assayed with a dual-luciferase assay system according to the manufacturer's instruction (Biotium Inc., Hayward, CA, USA). Results are expressed as luciferase/*Renilla* ratios and represent the mean±S.D. of three experiments, each performed in triplicate.

#### Bodipy-Cyclopamine (BC) binding assay

Human Myc-DDK-tagged Smo WT or human Myc-DDK-tagged Smo D473H or mouse Flag-tagged Smo WT or Flag-tagged Smo-D477G mutant were transfected in HEK293T cells. Cells were washed in PBS supplemented with 0.5% fetal bovine serum, fixed in 4% paraformaldehyde in phosphate-buffered saline (PBS) for 10 min, and incubated for 2 h at 37 °C both in the same medium supplemented with Bodipy-Cyclopamine (5 nM) and the studied compounds. The cells were permeabilized with Triton X100 (Sigma) 0.2%. Dako Fluorescent mounting (Dako, Carpinteria, CA, USA) was used as mounting medium and Hoechst reagent for staining of the cell nuclei. Bodipy (green) and Hoechst (blue) signals were analyzed in three to four representative fields per coverslip (× 20 magnification, 1000 cells/field). Data were expressed as percentage of BC incorporation observed with BC alone.^95^

#### mRNA expression analysis

Total RNA was isolated with Trizol (Invitrogen/Life Technologies, Carlsbad, CA, USA) and reverse transcribed with SensiFAST cDNA Synthesis Kit (Bioline Reagents Limited, London, UK). Quantitative real-time PCR (Q-PCR) analysis of *Gli1, Gli2, Ptch1, Bmp2, Pfkfb3, CycD1, CycD2, Pcna, Oct4, Nanog, β-2 microglobulin, β-actin and HPRT* mRNA expression was performed on each cDNA sample using the VIIA7 Real Time PCR System employing Assay-on-Demand Reagents (Life Technologies). A reaction mixture containing cDNA template, SensiFAST Probe Lo-ROX Kit (Bioline Reagents Limited) and primer probe mixture was amplified using FAST Q-PCR thermal cycler parameters. Each amplification reaction was performed in triplicate and the average of the three threshold cycles was used to calculate the amount of transcript in the sample (using SDS version 2.3 software). mRNA quantification was expressed, in arbitrary units, as the ratio of the sample quantity to the quantity of the calibrator. All values were normalized with two endogenous controls, *β-2 microglobulin* or *β-actin* and *HPRT*, which yielded similar results.

#### Cell proliferation and MB stem cells neurosphere-forming assay

Cell proliferation was evaluated by BrdU detection (Roche, Welwyn Garden City, UK). Briefly, after the BrdU pulse (24 h for GCPs) cells were fixed with 4% paraformaldehyde and permeabilized with 0.2% Triton X-100, and BrdU detection was performed according to the manufacturer's instructions. Nuclei were counterstained with Hoechst reagent. At least 500 nuclei were counted in triplicate, and the number of BrdU-positive nuclei was recorded. To determine the growth rate of viable cells, a trypan blue count was performed after a treatment period of 24–48–72 h with studied compound. For the neurosphere-forming assay, cells were plated at clonal density (1–2 cells/mm^2^) into 96-well plates and cultured in selective medium as previously described.^62^

#### Allograft experiment

A total of 2 × 10^6^ ASZ001 BCC cells were resuspended in an equal volume of 154CF medium and Matrigel (BD Biosciences, Heidelberg, Germany) and injected s.c. at the posterior flank of female NOD/SCID mice (Charles River Laboratories, Lecco, Italy), as previously described.^96^ Tumors were grown until a median size of ~200 mm^3^. Animals were randomly divided into two groups (*n*=6) and treated with solvent only (DMSO-Miglyol, 1:5) (Miglyol 812N by CREMER OLEO GmbH & Co. KG, Hamburg, Deutschland) or **12** in solvent (50 mg/kg) for 18 days. Tumor growth was monitored by measuring the size by caliper. Tumor volumes change was calculated by the formula length × width × 0.5 × (length+width).^97^ All animal experiments were approved by local ethics authorities.

#### Immunohistochemistry

For immunohistochemical staining tissues were formalin fixed and paraffin embedded. Sections were incubated with (1:100) rabbit monoclonal Ki67 antibody (Thermo Fisher Scientific, MA, USA) diluted in PBS. Detection was carried out with the mouse-to-mouse HRP (DAB) staining system (ScyTek Laboratories, Logan, UT, USA) accordingly to the manufacturer's instructions.

#### Statistical analysis

Statistical analysis was performed using StatView 4.1 software (Abacus Concepts Inc., Piscataway, NJ, USA). Statistical differences were analyzed with the Mann–Whitney *U*-test for nonparametric values, and a *P*<0.05 was considered significant. Results are expressed as mean±S.D. from an appropriate number of experiments (at least three biological replicas).

## Figures and Tables

**Table 1:**
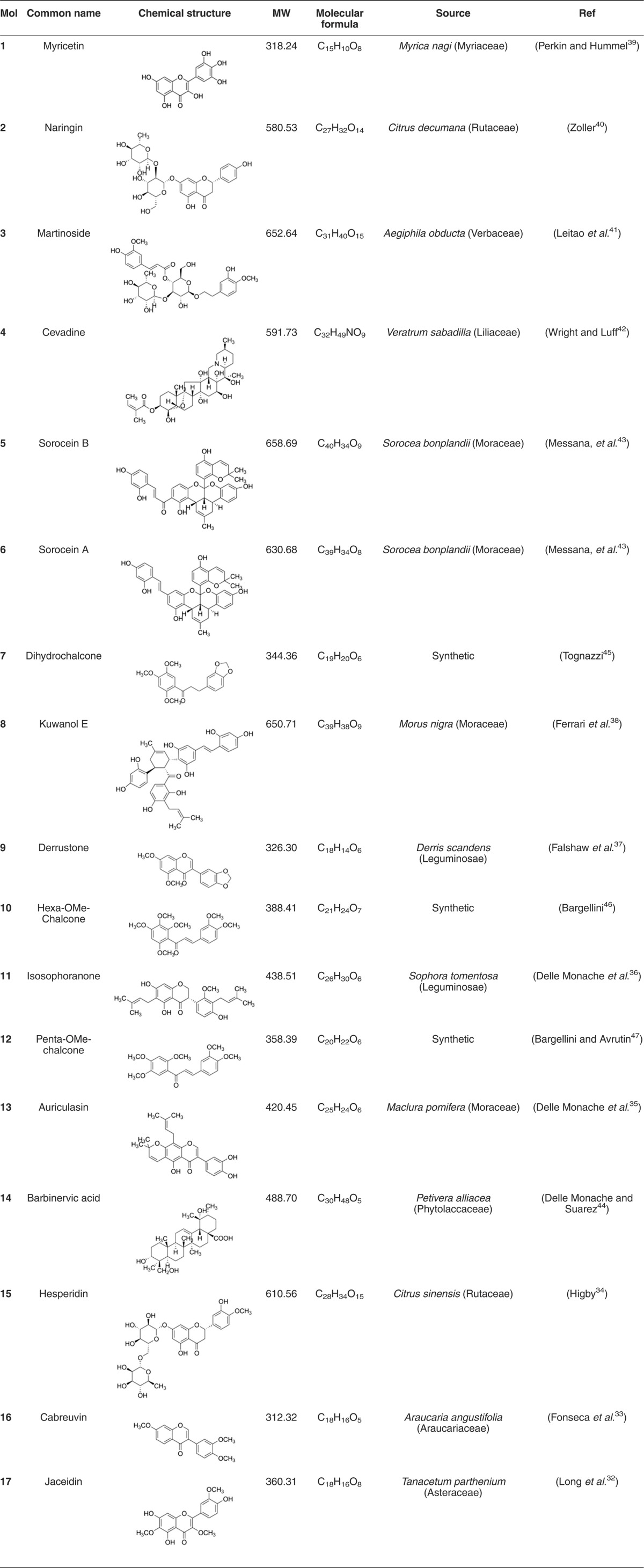
List of small molecules selected *in silico* as Smo inhibitors

## Abbreviations

Hh: Hedgehog
DMSO: dimethyl sulfoxide
SAG: Smoothened agonist
GCPs: Granule Cell Progenitors
BrdU: 5'-Bromo-2'-deoxy-Uridine
DMEM: Dulbecco's modified Eagle medium
FBS: fetal bovine serum
PBS: phosphate buffered saline
IF: immunofluorescence
BC: Bodipy-Cyclopamine
