# Supplementary material for: Inhibition of Hedgehog-dependent tumors and cancer stem cells by a newly identified naturally occurring chemotype
Source: Cell Death Dis. 2016 Sep 22;7(9):e2376–. doi: 10.1038/cddis.2016.195 (PMC5059851; doi:10.1038/cddis.2016.195)
Supplement: Supplementary Information [file cddis2016195x1.doc]

**Supporting Information**

**Chromatographic conditions for the purity check**

HPLC analytical separations were performed on a Waters 2690 Separation Module, equipped with a Rheodyne Model 8125 20-μl injector and a Model M486 programmable multi-wavelength detector (PDA). Chromatographic data were collected and processed using the Empower Chromatography Manager software.

**Compound 3**

Column: Zorbax Eclipse Plus C18, 1.8 μm (150 × 4.6 mm)

Eluent A) water/acetonitrile = 90:10 (v/v) + 1% formic acid. Eluent B) acetonitrile

Gradient elution: 0-25 min from A:B = 95:5 to 20% B; 25-30 min up to 40% B; 30-35 min up to 5% B

Flow rate: 0.5 ml/min

UV detection at 254 nm

Retention time (R_t_) = 28.13 min.

**Compound 4**

Column: Phenomenex Luna C18, 5 μm (250 × 4.6 mm)

Eluent: methanol/ammonium acetate 0.1 M = 60:40 (v/v) pH 4.5

Flow rate: 1.0 ml/min

UV detection at 254 nm

Retention time (R_t_) = 7.02 min.

**Compounds 5, 6, 8**

Column: Waters C18, 5.0 μm (150 × 4.6 mm)

Eluent A) water + 0.2% formic acid (pH 2.5). Eluent B) methanol

Gradient elution: 0-2 min from A:B = 90:10 to 30% B; 2-8 min up to 40% B; 8-13 min up to 50% B; 13-15 min A:B = 50:50; 15-23 min up to 70% B; 23-28 min A:B = 30:70; 28-38 min up to 80% B; 38-50 min A:B = 20:80; 50-55 min up to 95% B; 55-65 min A:B = 5:95; 65-60 min up to 90% B

Flow rate: 1.0 ml/min

UV detection at 330 nm

Retention times (R_t_) were as follows. For compound **6**, R_t_ = 32.83 min; for compound **5**, R_t_ = 45.51 min; for compound **8**, R_t_ = 45.90 min.

**Compounds 7, 9, 10-13, 16**

Column: Phenomenex Luna C18, 5.0 μm (250 × 4.6 mm)

Eluent A) water/acetonitrile = 95:5 (v/v). Eluent B) water/acetonitrile = 5:95 (v/v)

Gradient elution: for 0-5 min A:B = 50:50; 5-20 min up to 100% B; 20-25 min to 100% B

Flow rate: 1.0 ml/min

PDA detection at 200-400 nm

Retention times (R_t_) were as follows. For compound **12**, R_t_ = 7.12 min (at 330 nm); for compound **10**, R_t_ = 7.28 min (at 330 nm); for compound **7**, R_t_ = 8.20 min (at 330 nm); for compound **13**, R_t_ = 18.65 min (at 265 nm); for compound **11**, R_t_ = 18.79 min (at 295 nm); for compound **16**, R_t_ = 18.83 min (at 295 nm); for compound **9**, R_t_ = 18.87 min (at 295 nm).

**Compound 14**

Column: Phenomenex Luna C18, 5 μm (250 × 4.6 mm)

Eluent: methanol/0.1% H_3_PO_4_ aq. = 88:12 (v/v)

Flow rate: 1.0 ml/min

UV detection at 210 nm

Retention time (R_t_) = 3.83 min.

**Compound 17**

Column: Ascentis C18, 2.7 μm (150 × 2.1 mm)

Eluent A) water/acetonitrile = 95:5 (v/v). Eluent B) water/acetonitrile = 5:95 (v/v)

Gradient elution: 0 min A:B = 80:20; 0-20 min up to 100% B; 20-24 min 100% B; 24-25 min up to 20% B; 25-30 min A:B = 80:20

Flow rate: 0.3 ml/min

UV detection at 254 nm

Retention time (R_t_) = 14.21 min.

**Supplementary Figure legends**

**Figure S1.** **Inhibition of endogenous Hh signaling in NIH3T3 Shh-Light II cells.** (a-c) Effect of screened small molecules (used at 30 µM concentration) on Hh signaling showing no activity (a), mild (b) or high activity (c), in SAG-treated NIH3T3 Shh-Light II cells. Treatment time was 48 h, and normalization was against *Renilla* luciferase. Data show the mean ±SD of three independent experiments. **P*<0.05; ***P*<0.01 vs SAG.

**Figure S2.** **Predicted binding mode to Smo of moderately active Hh inhibitors**. Docking-based binding conformation of **6** (a), **7** (b), **8** (c), **9** (d) and **10** (e) within the antagonists’ site of Smo. The crystallographic structure of Smo encoded by PDB ID: 4O9R was used, and is showed as green cartoon. Residues within 5 Å from the ligands are showed as green lines. Residues involved in binding to well-known Smo antagonists are highlighted as green sticks. Small molecules are showed as cyan sticks. H-bond interactions are highlighted by black dashed lines.

**Figure S3**. **Compound 12 does not interact with D473**. Molecular docking results show that active Smo antagonists **12** binds in close proximity of the drug-resistance hot spot D473, but does not interact with the residue.

**Figure S4**. **Inhibition of Bodipy–Cyclopamine (BC) binding to whole cell expressing either Smo WT or Smo D477G by compound 12.** Competitive binding of Bodipy–Cyclopamine (BC) in HEK293T cells transiently transfected with mouse WT or mutant Smo D477G was conducted with various concentrations of compound **12**. BC binding (green) is visualized using fluorescence microscopy in a representative field (a). The concentrations-response curves express the percentage of BC incorporation observed after compound **12** treatment (b) in HEK293T cells transfected with mouse WT or mutant D477G Smo, respectively. Data show the mean ± SD of three independent experiments. **P*<0.05 vs CTR. Quantitative data are the average BC intensity from five independent fluorescence microscopy images.

**Figure S5**. **Compound** **12 inhibition of human BCC cell growth**. (a-c) TE354T BCC cells were treated with compound **12**, Vismodegib or DMSO only, as control. After the indicated times, a trypan blue count was performed to determine the growth rate (a-b). *Gli1* mRNA expression levels were determined by qRT–PCR after treatment of TE354T BCC cells with compound **12**, Vismodegib or DMSO (c). Results were normalized to endogenous control (*βactin* and *HPRT*). All data show the mean ± SD of three independent experiments. **P* < 0.05; ***P* < 0.01 versus DMSO (CTR).

**Figure S6. Compound** **12 inhibition of Hh signature in SuFu^-/-^ MEFs.** The graphs show the Hh target gene expression levels in SuFu^-/-^ treated for 48 h with compound **12** and DMSO, as a control. mRNA levels were determined by quantitative real-time PCR (qRT–PCR) normalized to

endogenous control (*β2-microglobulin* and *HPRT*). *Pfkfb3* gene was used as a negative control. Data show the mean ± SD of three independent experiments. **P* < 0.05 versus CTR.

**Table S1**. Biological activity data (IC_50_) and *in silico* score values of tested compounds **1**-**12**

| **mol** | **IC_50_ (µM)** | **Chemgauss4** | **Chemscore** | **Xscore (log*K*_d_)** | **MM-GBSA (kcal·mol^-1^)** |
| --- | --- | --- | --- | --- | --- |
| **1** | N.A. | -11.33 | 14.37 | 5.93 | -33.59 |
| **2** | N.A. | -11.53 | 5.08 | 7.38 | -68.23 |
| **3** | N.A. | -10.08 | 5.47 | 6.83 | -63.06 |
| **4** | N.A. | -10.89 | 4.81 | 8.44 | -56.70 |
| **5** | N.A. | -11.00 | 15.12 | 9.11 | -57.28 |
| **6** | 29.96 ± 1.93 | -13.64 | 21.65 | 9.52 | -62.13 |
| **7** | 38.47 ± 3.57 | -13.48 | 20.38 | 5.87 | -41.13 |
| **8** | 27.49 ± 2.67 | -14.46 | 23.92 | 8.96 | -54.87 |
| **9** | 24.12 ± 1.60 | -13.85 | 24.05 | 6.09 | -37.82 |
| **10** | 31.31 ± 2.29 | -14.43 | 22.42 | 5.8 | -42.06 |
| **11** | 22.56 ± 2.25 | -19.31 | 29.18 | 7.44 | -63.47 |
| **12** | 4.44 ± 0.43 | -15.69 | 27.49 | 6.08 | -45.48 |

N.A. Not Active

**Table S2.** Binding of compound **12** vs Vismodegib to both murine and human wild-type or drug-resistance Smo mutants in transiently transfected 293T cells, expressed as IC_50_ (μM)

| **Compound** | **Murine**  **Smo WT** | **Human**  **Smo WT** | **Murine**  **Smo D477G** | **Human**  **Smo D473H** | **Fold shift**  **murine Smo** | **Fold shift**  **human Smo** |
| --- | --- | --- | --- | --- | --- | --- |
| **12** | 45.62 | 24.50 | 56.71 | 22.68 | 1.243095134 | 0.925714286 |
| **Vismodegib** | 0.011 | 0.007624 | 1.14 | 10.45 | 103.6363636 | 1370.671563 |
