# Supplementary figures and images for: Inhibition of Hedgehog-dependent tumors and cancer stem cells by a newly identified naturally occurring chemotype
Source: Cell Death Dis. 2016 Sep 22;7(9):e2376–. doi: 10.1038/cddis.2016.195 (PMC5059851; doi:10.1038/cddis.2016.195)

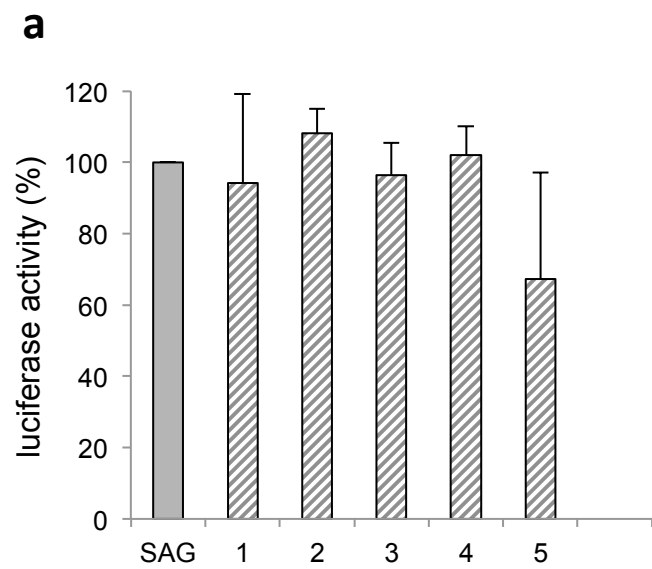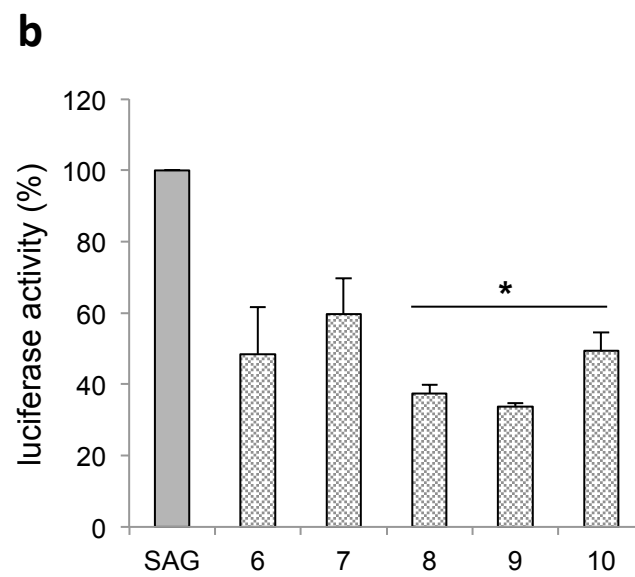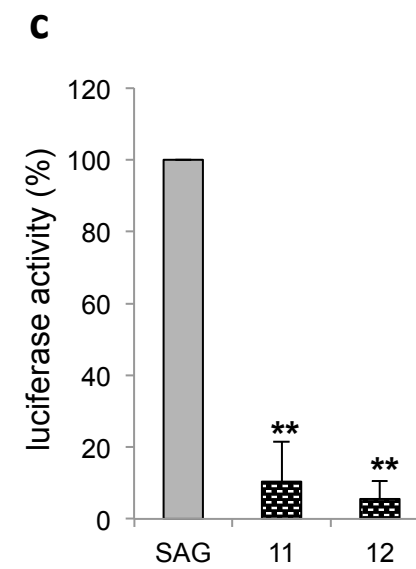

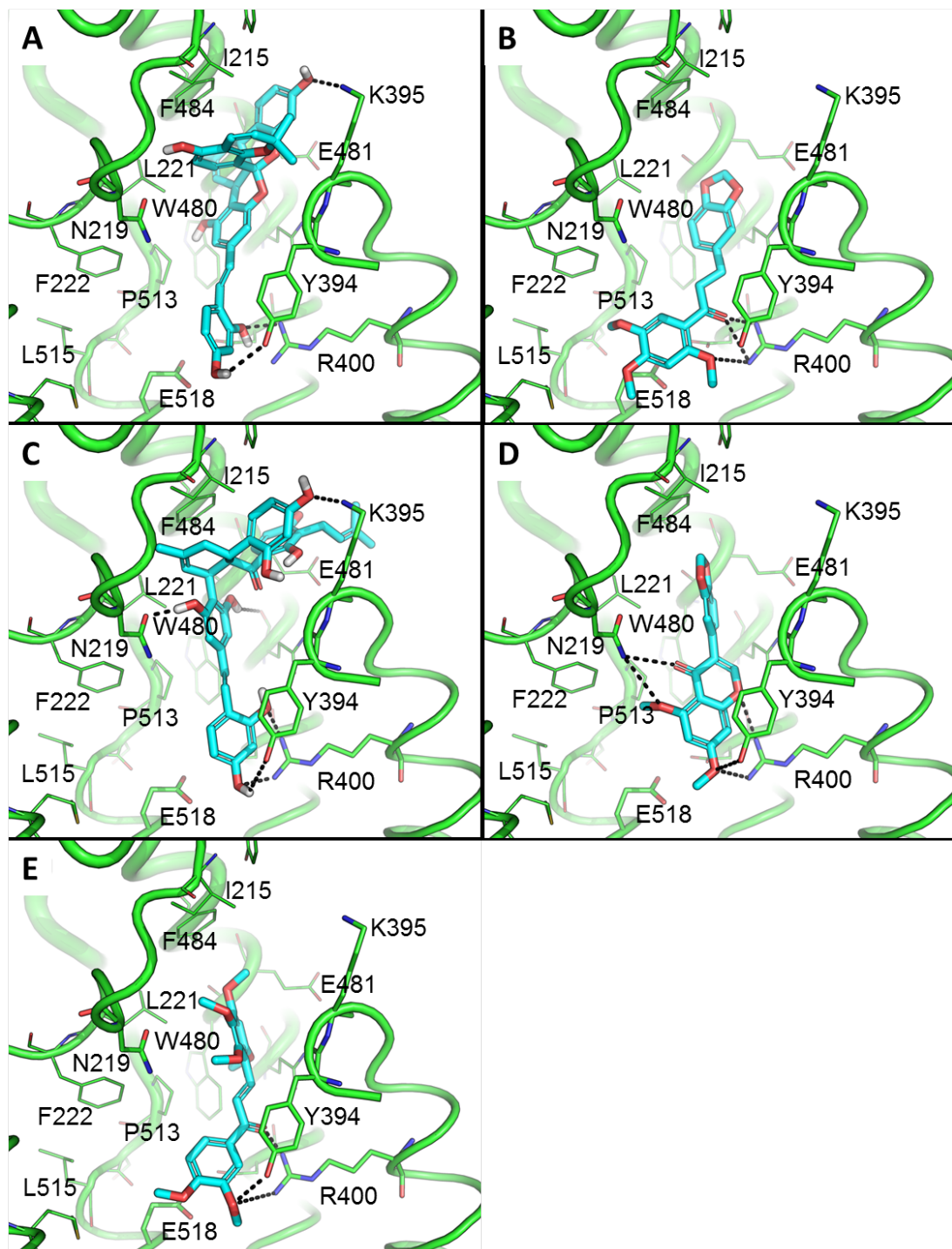

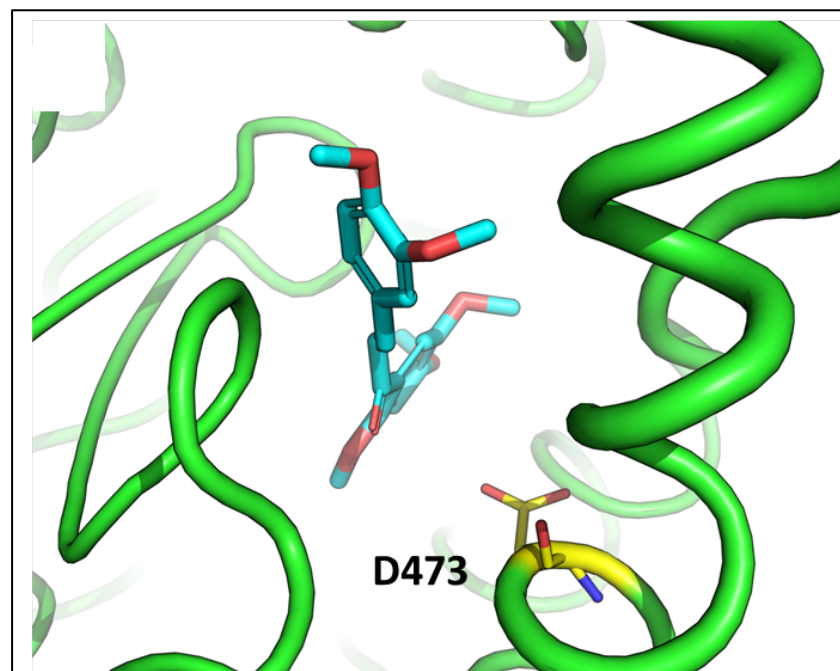

**a**

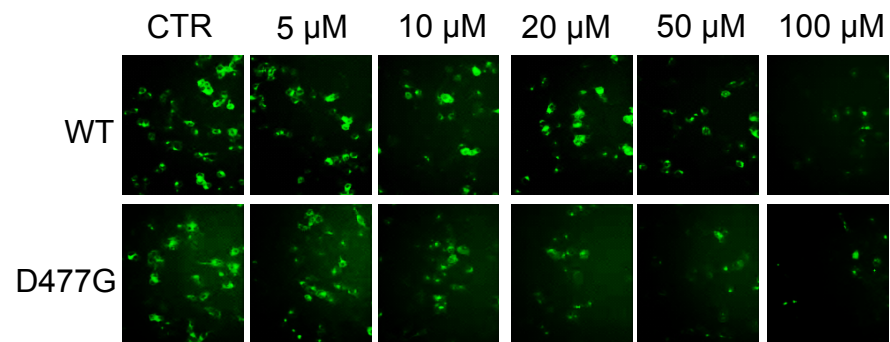

**b**

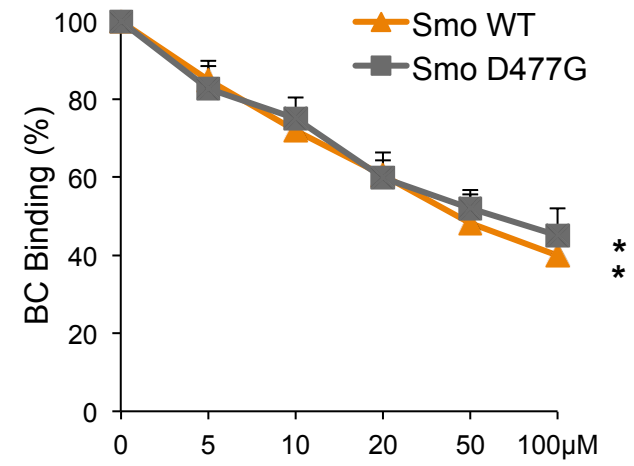

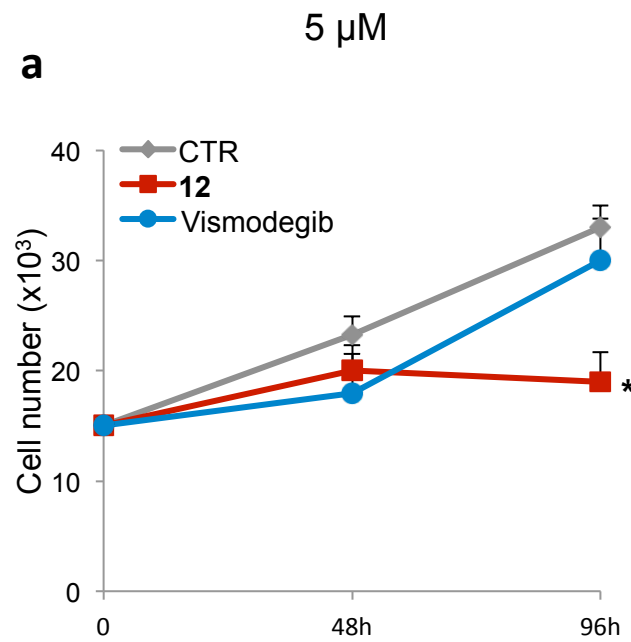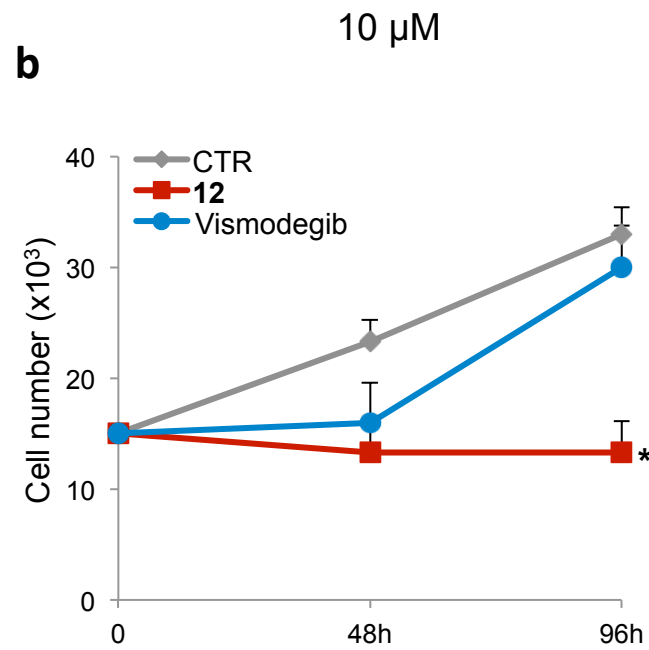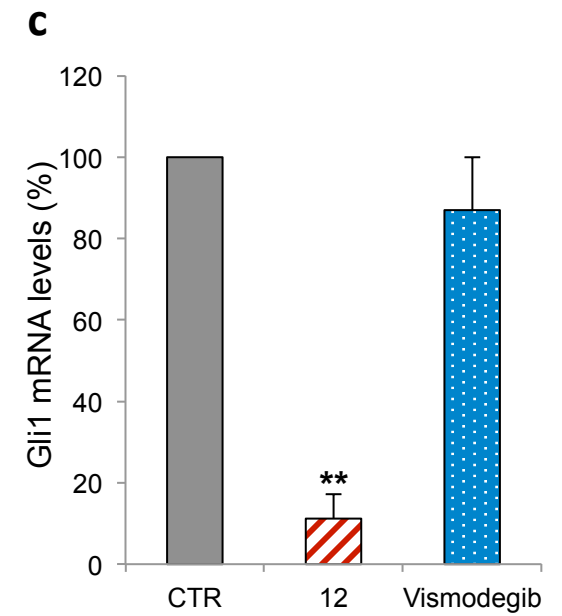

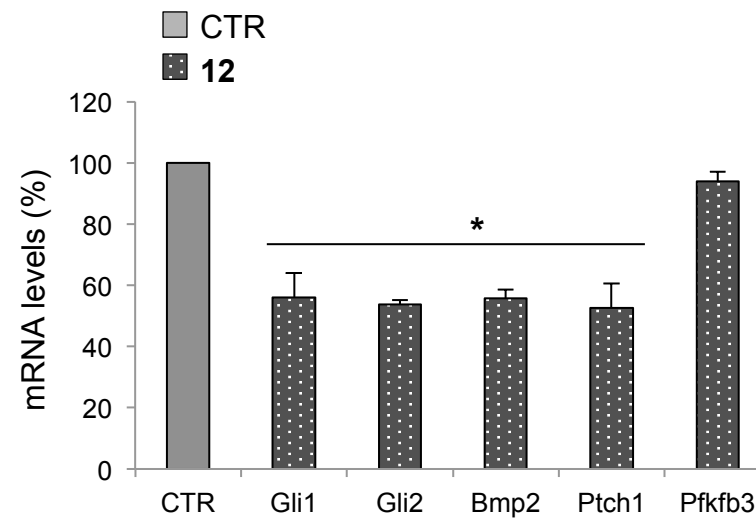

Supplement: Supplementary Figures [file cddis2016195x2.pdf]
